# A Randomized Controlled Trial Comparing Ferric Carboxy-Maltose With Iron Sucrose Complex for Postpartum Iron-Deficiency Anemia

**DOI:** 10.7759/cureus.93076

**Published:** 2025-09-23

**Authors:** Chanderdeep Sharma, Manupriya Sharma, Varun Kapoor, Tanu Verma, Anjali Soni, Suresh Verma

**Affiliations:** 1 Obstetrics and Gynecology, All India Institute of Medical Sciences, Bilaspur, Bilaspur, IND; 2 Pathology, All India Institute of Medical Sciences, Bilaspur, Bilaspur, IND; 3 Obstetrics and Gynecology, Department of Health and Family Welfare, Government of Himachal Pradesh, Mandi, IND; 4 Obstetrics and Gynecology, Dr. Radha Krishnan Government Medical College, Hamirpur, IND; 5 Obstetrics and Gynecology, Dr. Rajendra Prasad Government Medical College, Tanda, IND

**Keywords:** ferric carboxy maltose, iron-deficiency anemia, iron sucrose-complex, postpartum, pregnancy

## Abstract

Objective: To determine the efficacy of parenteral iron carboxy-maltose (FCM) versus iron sucrose complex (ISC) for the treatment of postpartum iron-deficiency anemia (IDA).

Methods: A randomized controlled trial was conducted among postpartum women with IDA. Primary outcome was to compare the efficacy of FCM versus ISC in the improvement of hemoglobin (Hb), RBC indices (mean corpuscular Hb (MCH), mean corpuscular volume (MCV), mean corpuscular Hb concentration (MCHC)), and iron stores (serum iron, serum ferritin, percentage saturation of iron, and total iron binding capacity). Secondary outcomes were side-effect profile and maternal complications. At enrolment, 500 women were randomized to two groups. Demographic and clinical data were collected at enrolment and four weeks later.

Results: Women receiving FCM had statistically significantly higher Hb levels (g/dl) (11.4 (10.3-12.5) vs. 11 (9.1-12.9), p<0.001), better RBC indices (MCV (fl); 96.5 (87.5-105) vs. 92 (77-107), p<0.001, MCH (pg/cell); 32 (27-37) vs. 30 (25-35), p<0.001, MCHC (g/dl); 36 (31-41) vs. 34 (30-38), p=0.001) and better iron stores (serum ferritin (µg/l); 290 (273-307) vs. 229 (213-245), p<0.001, and percentage saturation of iron (%); 24.5 (16.5-32.5) vs. 21 (16-26), p<0.001). However, there was no difference regarding serum iron (p=0.513) and total iron binding capacity (p=0.298). ISC had poor compliance as 23 (9.2%) women did not come for repeated doses. Secondary outcomes (side-effect profile (p=0.797) and maternal complications (p=0.176)) were comparable among women.

Conclusion: Ferric carboxymaltose has better efficacy and compliance for the treatment of postpartum iron-deficiency anemia as compared to iron-sucrose complex, with a similar side effect profile. We recommend that women with postpartum iron deficiency anemia (Hb 7-11 g%) receive at least a single parenteral FCM (1000 mg) before discharge from the hospital as a standard policy, especially in low- and middle-income countries.

## Introduction

Anemia is a global health problem [1]. Women, especially during pregnancy and the post-partum (PP) period, have a very high incidence of anemia (34-50%) [2]. PP anemia affects 50% of women in developed countries and 80% in developing countries [2].

The most common cause (>50%) of this anemia is iron deficiency anemia (IDA) due to antepartum iron deficiency, iron demands from the fetus, and peripartum blood loss [3]. PP-IDA may have long-term health implications for the mother and her infant. Mothers with low iron stores at the time of delivery and following childbirth may experience fatigue, altered cognition, and depressive symptoms [4]. These alterations in the mother’s emotional and cognitive functioning may, in turn, affect her interactions with the infant and negatively affect health-related quality of life. Iron replenishment improves both symptoms of fatigue and depression [5]. If iron stores are not restored soon after childbirth, the negative consequences of PP-IDA may continue through other stages of the reproductive cycle (particularly in women with a high prevalence of anemia and short inter-pregnancy intervals), leading to continued adverse maternal and infant outcomes [4,5].

WHO has defined post-partum anemia as hemoglobin (Hb) of <12 g% [6]. Current WHO guidelines recommend, “Oral iron supplementation, either alone or in combination with folic acid supplementation, may be provided to post-partum women for 6-12 weeks following delivery for reducing the risk of anemia in settings where gestational anemia is of public health concern” [2]. Oral iron has been the mainstay of treatment for PP-IDA [2-5]. However, about 40% of patients on oral iron experience gastrointestinal adverse effects such as vomiting, constipation, diarrhea, and abdominal pain, thereby leading to reduced compliance and persistent anemia [7]. Additionally, oral iron requires a prolonged duration of therapy to correct anemia and replenish iron stores [7]. However, compliance with oral iron therapy after childbirth is deficient (20.8%) [8].

Hence, parenteral iron could be a better alternative to oral iron in patients who cannot tolerate oral iron, are non-compliant, or need rapid restoration of iron stores. Parenteral iron helps restore iron stores faster and more effectively, with ease of administration, less frequent dosing, lesser side effects, and prolonged benefits. It has even reduced the need for blood transfusions [6]. This situation is ideal in low- and middle-income countries (LMICs) where the loss to follow-up is common for various reasons, especially in the post-partum period [8].

Of all available parenteral iron preparations, ferric carboxy-maltose (FCM) and iron sucrose complex (ISC) are two commonly preferred regimens [6]. To the best of our knowledge, no study has been done comparing these two agents for treating PP-IDA in the modern era of evidence-based medicine. Hence, we planned this study to compare the efficacy, side effects, and compliance of these two parenteral formulations (FCM and ISC) for treating PP-IDA.

## Materials and methods

This randomized trial was conducted to compare the parenteral iron (FCM versus ISC) for the treatment of post-partum IDA in the Department of Obstetrics and Gynecology, Dr. Rajendra Prasad Government Medical College, Kangra at Tanda (HP), India, a tertiary care teaching and training hospital. Institutional ethics committee approval was taken prospectively (letter no. HFW-HDRPGMC/Ethics/2019/125 dated 09.01.2019). Recruitment took place from January 2019 to December 2019. The study was also registered prospectively in the Clinical Trial Registry of India (CTRI) www.ctri.nic.in (registration number CTRI/2018/08/015366, date 16-08-2018).

All women with anemia (Hb <9g%) were approached for enrolment. Inclusion criteria were women with Hb between 7-9 g% with confirmed IDA (confirmed by red blood cell indices, peripheral smear and iron studies; mean corpuscular volume (MCV) <75fl, peripheral smear microcytic hypochromic, iron studies; serum ferritin (<20ug/l), total iron binding capacity (<300ug/dl), percentage saturation of iron (<16%), and serum iron (<60ug/100ml)). Exclusion criteria were hemodynamically unstable women, severe anemia (Hb <7 gm%), anemia due to other causes like thalassemia, sickle cell anemia, thalassemia, aplastic anemia, megaloblastic anemia, anemia due to liver disease, and kidney disease, known history of allergy to injection iron, blood transfusion in the antenatal period, congestive cardiac failure, sepsis known case of hemochromatosis or hepatitis (A, B, C, D, E).

Consolidated Standards of Reporting Trials (CONSORT) guidelines were strictly followed throughout the trial. Written informed consent was taken from all the participating women. After careful assessment by a senior consultant, 500 post-partum women were randomized based on a computer-generated random number table to either of the two groups: group 1 (FCM; Orofer FCM 1K Emcure Pharmaceuticals Ltd., Pune, India) and group 2 (ISC; Orofer S 200, Emcure Pharmaceuticals). Allocation concealment was done in sealed, opaque envelopes opened just before administering parenteral iron. They were locked in a box in the ward. Randomization sequence was computer-generated in blocks of four or eight. The primary investigator (CS) enrolled participants. Sealed opaque envelopes were opened by the resident doctor on duty. Two hundred fifty women were included in each group.

The study's primary outcome was to compare the efficacy of FCM and ISC in treating PP-IDA. The efficacy of the treatment was determined by improvement in Hb, red blood cell indices (MCV, mean corpuscular Hb (MCH), mean corpuscular Hb concentration (MCHC)), and iron studies (serum ferritin, serum iron, total iron binding capacity (TIBC) and percentage saturation of iron). Secondary outcomes were side effect profile (nausea, vomiting, fever, arthralgia, headache, injection site thrombophlebitis, hypotension, and anaphylactic reaction) and maternal complications (sub-involution of uterus, lactation failure, puerperal sepsis and subsequent requirement of blood transfusion).

The dosage of parenteral iron was calculated by the following formulae: 2.4 × body weight (in kgs.) × Hb deficit (D) + 500 mg (D = Hb deficit (Desired Hb - actual Hb) and 500 mg additional to replenish the body iron stores (dose rounded off to nearest 100 mg)). A total of 250 women were randomized to group 1; injection FCM was administered by intravenous (IV) drip infusion (as per dose calculation, a maximum single dose of 1000 mg diluted in 100 mL sterile 0.9% NaCl solution over 30 min not more than once a week, if women required higher dose (>1000 mg, then the additional dose was given next week)). In group 2, 250 women were administered injection ISC by IV drip infusion (as per dose calculation, a maximum single dose of 200 mg diluted in 100 mL sterile 0.9% NaCl solution over 30 min, to be repeated on alternate days with a maximum of 600 mg of ISC per week). Women who requested discharge from the hospital but required multiple iron doses (both FCM and ISC) were given the remaining doses on an outpatient basis. All women were observed for any side effects like headache, nausea, diarrhea, vomiting, pain, muscle pain, cardiovascular collapse and burning at the injection site, rigor, fever, hypotension and hypertension, tingling sensation, itching, or any other side effects for one hour after injection. Women were closely monitored for any side effects. Women were followed up after four weeks, enquired about any side effects, and were investigated (Hb, MCV, MCH, MCHC, iron studies (serum ferritin, serum iron, TIBC, percentage saturation of iron)). They were also assessed for sub-involution of the uterus, lactation failure, puerperal sepsis, and the need for blood transfusion. Any complications reported were recorded as per the study protocol.

The sample size was calculated based on assuming that mean improvement in Hb with FCM would be 2.59±0.94 g/dl and mean improvement with ISC 2.23 g/dl at 95% confidence interval and α error of 0.05, power of 80% sample size came out to be 205 for each group (similarly sample size was estimated for each of the variable in the primary outcome i.e. Hb, RBC indices (MCV, MCH, MCHC) and iron studies (serum ferritin, serum iron, TIBC, percentage saturation), and largest size was selected i.e. 250 women in each group). As women (in group 2) required repeat injections over a span of period (as per dose calculation, dose administration 600 mg per week) so assuming an attrition of 20% (previous unpublished data from our institute), 250 women were allocated to each group (total 500 women). Statistical analysis was carried out based on intention-to-treat. Data were entered into Excel (Microsoft, Redmond, WA, USA) and analyzed using Epi-Info 7 (CDC, Atlanta, GA, USA). The corresponding author validated the accuracy of the database. Analysis was performed using parametric and non-parametric tests whenever considered appropriate. The Kolmogorov-Smirnov test assessed the normality of distribution. Continuous data were analyzed using the t-test (normal distribution) and Mann-Whitney U test (non-normal distribution), whereas Fisher's exact test analyzed categorical variables. P<0.05 was considered statistically significant.

## Results

**Figure 1:**
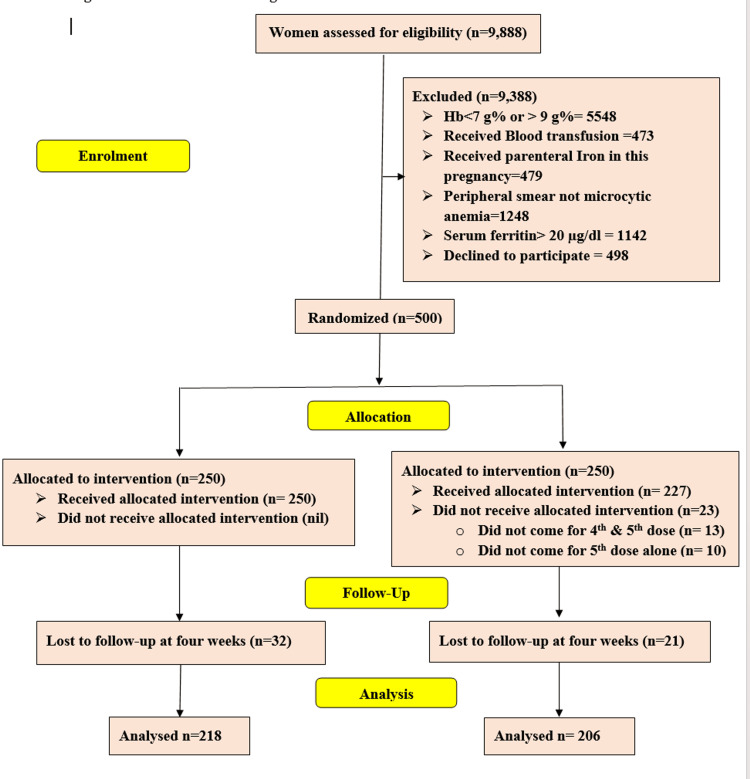
CONSORT flow chart CONSORT; Consolidated standards of reporting trials

Figure 1 shows the CONSORT flow chart of women after enrolment. During the study period, 500 women were randomized to either of the study groups. In group 2 (ISC), 23 women did not receive complete allocated treatment (13 women did not come for the fourth and fifth doses, and 10 did not come for the fifth dose alone). Additionally, 32 women in group 1 and 21 in group 2 were lost to follow-up at four weeks for re-assessment. So, the number of women finally analyzed in two groups was 218 and 206, respectively, as shown in Figure 1.

Table 1 shows the mean characteristics of all women. There was no statistically significant difference between the groups concerning age, BMI, parity, mode of delivery, pre-treatment Hb, RBC indices (MCV, MCH, MCHC), iron studies (serum ferritin, serum iron, TIBC, and percentage saturation of iron), post-partum hemorrhage, birth weight of neonate, sex of neonate. However, the total iron requirement (mg) (group 1 vs. 2; 873 (747-999) vs. 860 (676-1044), p=0.029) was significantly more in the FCM group (group 1). Also, total number of doses was significantly more in group 2 (ISC) (median (IQR); 5 (4-5) vs. 1 (1-2), p<0.001).

**Table 1:** Mean characteristics of women in the study FCM: ferric carboxymaltose; ISC: iron sucrose complex; FCM: ferric carboxy-maltose; ISC: iron sucrose complex; CS: cesarean section; Hb: hemoglobin; MCV: mean corpuscular volume in femtoliter; MCH: mean corpuscular hemoglobin in picograms; MCHC: mean corpuscular hemoglobin concentration in gram per deciliter; µg/l: microgram per liter; µg/dl: microgram per deciliter; TIBC: total iron binding capacity; mg: milligram; #: post-partum hemorrhage within 24 h of delivery; *: median (inter-quartile range for non-normal distribution); P-value<0.05 significant

	Group 1 FCM (n=218)	Group 2 ISC (n=208)	P-value
Age (years)*	27 (19-35)	27 (19-35)	0.750
Body Mass Index (Kg/m^2^) *	24.8 (23-26)	25.1 (22.8-26.7)	0.184
Primae gravida (n)	102	90	0.496
Multi gravida (n)	114	116	0.497
Vaginal delivery (n)	136	131	0.920
Operative vaginal delivery (n)	3	-	0.249
Cesarean Section (CS)(n)	76	78	0.614
Hb (g/dl) *	8 (7.1-8.9)	8 (7-9)	0.202
MCV (fl)	68 (56-80)	69 (56-82)	0.103
MCH (pg)	23 (17-29)	23 (18-28)	0.355
MCHC (g/dl)	27 (21-33)	26 (20-32)	0.908
Serum ferritin (µg/l)	12 (8-16)	12 (6-18)	0.805
Serum Iron (µg/dl)	28 (20-36)	27 (18-37)	0.897
TIBC (µg/dl)	546 (355-737)	546 (349-743)	0.709
Percentage Saturation of iron (%)	12 (9-15)	12 (8-16)	0.514
Total iron requirement (mg)	873 (747-999)	860 (676-1044)	0.029
Primary post-partum hemorrhage (n)^#^	6	8	0.456

**Figure 2:**
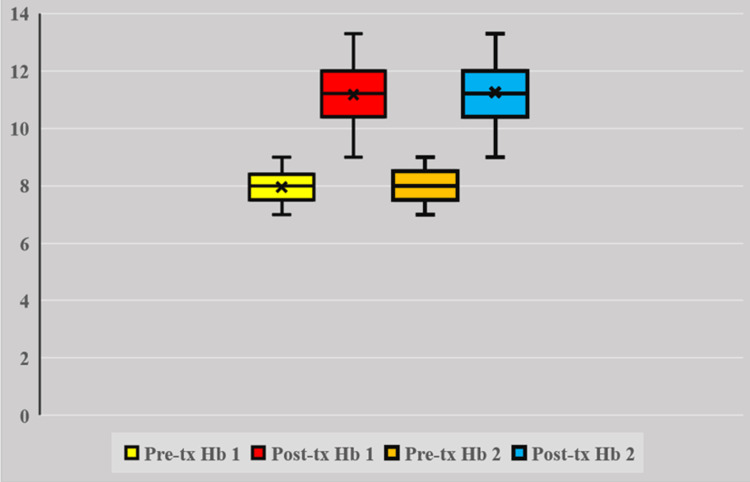
Hemoglobin levels (g/dl) pre- and post-treatment in women Pre-tx Hb 1: pre-treatment hemoglobin levels in group 1 (ferric carboxy-maltose); Post-tx Hb 1: post-treatment hemoglobin levels in group 1 (ferric carboxy-maltose); Pre-tx Hb 2: pre-treatment hemoglobin levels in group 2 (iron sucrose complex); and Post-tx Hb 2: post-treatment hemoglobin levels in group 2 (iron sucrose complex)

**Figure 3:**
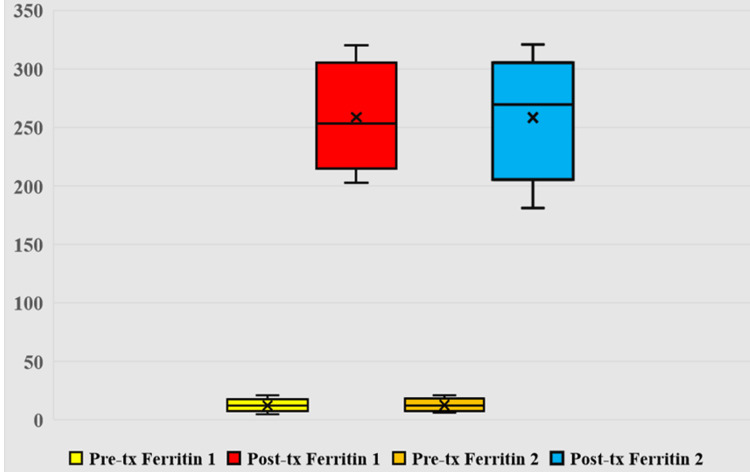
Serum ferritin levels (mcg/L) pre- and post-treatment in women Pre-tx Ferritin 1: pre-treatment serum ferritin levels in group 1 (ferric carboxy-maltose); Post-tx Ferritin 1: post-treatment serum ferritin levels in group 1 (ferric carboxy-maltose); Pre-tx Ferritin 2: pre-treatment serum ferritin levels in group 2 (iron sucrose complex); and Post-tx Ferritin 2: post-treatment serum ferritin levels in group 2 (iron sucrose complex)

Women receiving FCM had statistically significantly higher Hb levels (g/dl) (11.4 (10.3-12.5) vs. 11 (9.1-12.9), p<0.001) as shown in Figure 2, better RBC indices (MCV (fl); 96.5 (87.5-105) vs. 92 (77-107), p<0.001, MCH (pg); 32 (27-37) vs. 30 (25-35), p<0.001, MCHC (g/dl); 36 (31-41) vs. 34 (30-38), p=0.001) and better iron stores (serum ferritin (µg/l); 290 (273-307) vs. 229 (213-245), p<0.001, as shown in Figure 3, and percentage saturation of iron (%); 24.5 (16.5-32.5) vs. 21 (16-26), p<0.001). However, there was no difference regarding serum iron (µg/dl) (group 1 vs. 2; 165 (153-177) vs. 165 (153-177) p=0.513), and total iron binding capacity (µg/dl) (group 1 vs. 2; 339 (268-410) vs. 350 (268-410), p=0.298). These statistical differences were still significant after the analysis of variance test (ANOVA) was used to account for the initial difference in the requirement of iron dose in the two groups, as shown in Table 2.

**Table 2:** Primary outcomes of the study FCM: ferric carboxy-maltose; ISC: iron sucrose complex; OR: odds ratio; CI: confidence interval; *: Analysis of variance adjusted for total iron requirement; Hb: Hemoglobin; MCV: mean corpuscular volume; MCH: mean corpuscular hemoglobin; MCHC: mean corpuscular hemoglobin concentration; TIBC: total iron binding capacity; P-value<0.05 significant; Box-cox transformation is used to construct the confidence intervals due to highly skewed non-parametric data

	Group 1 FCM (n=218)	Group 2 ISC (n=208)	OR [95% CI]	P-value	P-value ANOVA^*^
Post treatment Hb (gm/dl)	11.4 (10-12.5)	11 (9.1-12.9)	(0.176-0.551)	<0.001	<0.001
Post treatment MCV (fl)	96.5 (87-105)	92 (77-107)	(0.209-0.583)	<0.001	<0.001
Post treatment MCH (pg)	32 (27-37)	30 (25-35)	(0.258-0.630)	<0.001	<0.001
Post treatment MCHC (g/dl)	36 (31-41)	34 (30-38)	(0.124-0.501)	0.003	0.001
Post treatment serum ferritin (µg/l)	290 (273-307)	229 (213-245)	(1.778-1.922)	<0.001	<0.001
Post treatment serum iron (µg/dl)	165 (153-177)	165 (153-177)	(0.127-0.254)	0.820	0.513
Post treatment TIBC (µg/dl)	339 (268-410)	350 (268-432)	(0.292-0.089)	0.111	0.298
Post treatment % saturation of iron	24.5 (16-32.5)	21 (16-26)	(0.225-0.599)	<0.001	<0.001

Secondary outcomes were comparable among women (side effects profile (n); 36 vs. 37, p=0.797 (vomiting (n) 1/218 vs. 4/208, p=0.335, fever (n); 29/218 vs. 22/208, p=0.407, injection site thrombophlebitis (n); 4/218 vs. 9/208, p=0.081 and arthralgia (n); 2/218 vs. 2/208, p=1.000) and maternal complications (sub-involution of uterus (n); 9 out of 218 vs. 4 out of 208, p=0.081 and lactation failure (n); 29/218 vs. 22/208, p=0.407). No women in the study had an anaphylactic reaction to either of the parenteral iron preparations (FCM or ISC). No women subsequently required a blood transfusion.

## Discussion

We observed statistically significant improvement in the Hb levels (p<0.001), RBC indices (MCV (p<0.001), MCH (p<0.001), and MCHC (p=0.001)) and iron stores (serum ferritin (p<0.001) and percentage saturation of iron (p<0.001)), with FCM for the treatment of PP-IDA, as compared to ISC in postpartum women with moderate IDA. Both these drugs have been extensively studied separately [9,10]. However, there needs to be direct comparison of FCM and ISC on PP-IDA. To the best of our knowledge, to date no randomized trial has been done comparing these two drugs for treating PP-IDA.

Sharma N et al. [11] did a comparative study of 120 women, observing FCM to be superior to ISC in improving Hb levels (p=0.000) and serum ferritin levels (p=0.000) after two weeks of treatment. However, sample size selected was very small (n=120). Small sample size in clinical trials can lead to limited generalizability, increased variability, reduced statistical power, and a higher risk of false results. Furthermore, in this non-randomised trial the follow-up period was also very short (only two weeks), which may inaccurately depict the improvement in Hb levels as the minimum time for improvement of Hb levels is one to three weeks [6].

Pfenniger A et al. [12] conducted a retrospective cohort study involving 210 women to compare the safety and efficacy of intravenous FCM with ISC for the treatment of postpartum anemia. They observed both drugs to be safe, effective, and rapidly normalizing Hb after eight days of treatment. Being a retrospective cohort study, the study design had inherent disadvantages of bias (selection bias, observer bias and recall bias), limited control over data collection (incomplete or missing data: important information may not be available or may be missing from existing records), inconsistent data collection, poor data quality and low internal and external validity. Also, their period of follow-up was very short, i.e., only eight days.

Jose A et al. [13] conducted an RCT to evaluate the efficacy and safety of intravenous FCM with ISC for the treatment of iron deficiency anemia in pregnancy (n=100) in women with moderate to severe iron deficiency anemia. They observed significant improvement in Hb levels at 12 weeks in the FCM group as compared to the ISC group (29 g/L vs 22 g/L; p value < 0.01). They hypothesized that the convenient dosing schedule of FCM will have better compliance than that of ISC. Even though they had a robust study design as an RCT, their trial had a much smaller sample size as compared to our study (n=100 vs. 500).

Our findings imply that FCM has better efficacy for treating PP-IDA than ISC. Additionally, there is an inherent advantage of using FCM, i.e., high single dose infusion (as per body weight) in a relatively shorter time (20 minutes). Meanwhile, ISC needs multiple dosing, low single dose infusion (200 mg), and limited weekly maximum dose (not > 600 mg). Due to the need for repeat parenteral administration requiring multiple visits, poor compliance (9.2%; 23 out of 250) is a significant concern for its use in postpartum women. Side effect profiles (p=0.797) and maternal complications (p=0.176) were comparable in the two groups. Both drugs were safe (no woman had an anaphylactic reaction or required a blood transfusion subsequently).

As per WHO guidelines, oral iron is the first line of treatment of PP-IDA [1]. However, its use has several limitations, i.e., poor tolerability, prolonged duration of treatment, and poor absorption [9,10]. Even increasing the dose of oral iron does not improve efficacy [10]. Parenteral FCM can easily overcome all these limitations.

FCM is a non-dextran iron complex that consists of a ferric hydroxide core stabilized by a carbohydrate shell. The design of the macromolecular ferric hydroxide carbohydrate complex permits guarded delivery of iron to the cells of the reticuloendothelial system and subsequent delivery to the iron-binding proteins, ferritin, and transferrin, with negligible risk of large amounts of ionic iron being released into the serum with low immunogenic potential [9,10]. Parenteral FCM corrects IDA faster and in a single dose (as per body weight), with fewer side effects, better compliance, bio-availability, favorable dosing schedule (usually single dose), thereby requiring less patient-provider interaction, and low immunogenic potential [9,10]. However, we did not assess cost-effectiveness as both drugs are freely available to all patients under the national scheme.

We recommend that women with moderate (Hb=7-9g%) PP-IDA should receive parenteral FCM (1000 mg) as a preferred treatment before discharge from the hospital as a standard policy. It will not only correct PP-IDA rapidly but also improve iron stores for future reproductive cycles. This statistically significant improvement of Hb, RBC indices, and iron stores can significantly reduce maternal, infant, and subsequent maternal morbidity and mortality for future pregnancies.

The main strength of our study is a robust study design (randomized controlled trial) with adequate sample size (500 women) and power (80%). We included women after both vaginal and caesarean delivery, thereby increasing the generalisability of our findings.

One major limitation of our study was that all women received open-label treatment. As the dosing schedules of these two agents were different, it was not considered ethically justified to call women to give placebo injections repeatedly. However, this lack of blinding can have minimal bias, if any, on Hb, RBC indices, and iron stores. Another major limitation is the cost of parenteral FCM (as both drugs were freely available in our institute so cost-effectiveness could not be evaluated).

A large, multi-centric, cost-effective trial is the urgent need of the hour, especially regarding the long-term benefits of correcting anemia, a global health problem, to routinely incorporate this agent into national policy guidelines for postpartum women.

## Conclusions

To conclude, parenteral FCM has better efficacy and compliance for treating postpartum iron deficiency anemia than parenteral ISC, with a similar side effect profile. The safety profile of both these agents is comparable. We recommend that women with moderate postpartum iron deficiency anemia (Hb; 7-11 g%) receive at least a single parenteral FCM (1000 mg) before discharge from the hospital as a standard policy.
